# Neurological Signs at the First Psychotic Episode as Correlates of Long-Term Outcome: Results From the AESOP-10 Study

**DOI:** 10.1093/schbul/sbaa089

**Published:** 2020-07-13

**Authors:** Naika P Ferruccio, Sarah Tosato, Julia M Lappin, Margaret Heslin, Kim Donoghue, Annalisa Giordano, Ben Lomas, Ulrich Reininghaus, Adanna Onyejiaka, Raymond C K Chan, Tim Croudace, Peter B Jones, Robin M Murray, Paul Fearon, Gillian A Doody, Craig Morgan, Paola Dazzan

**Affiliations:** 1 Department of Psychosis Studies, Institute of Psychiatry, Psychology and Neuroscience, King’s College London, London, UK; 2 Department of Neuroscience, Biomedicine and Movement Sciences, Section of Psychiatry, University of Verona, Verona, Italy; 3 School of Psychiatry, Faculty of Medicine, University of New South Wales, Sydney, Australia; 4 Department of Health Service and Population Research, Institute of Psychiatry, Psychology and Neuroscience, King’s College London, London, UK; 5 Department of Addictions, Institute of Psychiatry, Psychology and Neuroscience, King’s College London, London, UK; 6 Department of Psychiatry, University of Nottingham, Nottingham, UK; 7 Department of Public Mental Health, Central Institute of Mental Health, Medical Faculty Mannheim, University of Heidelberg, Heidelberg, Germany; 8 Neuropsychology and Applied Cognitive Neuroscience Laboratory, CAS Key Laboratory of Mental Health, Institute of Psychology, Chinese Academy of Sciences, Beijing, China; 9 School of Health Sciences, University of Dundee, Dundee, UK; 10 University of Cambridge, and Cambridgeshire and Peterborough NHS Foundation Trust, Cambridge, UK; 11 Discipline of Psychiatry, School of Medicine, Trinity College, Dublin, Ireland; 12 Department of Psychological Medicine, Institute of Psychiatry, Psychology and Neuroscience, King’s College London, London, UK; 13 National Institute for Health Research (NIHR) Mental Health Biomedical Research Centre at South London and Maudsley NHS Foundation Trust and King’s College London, London, UK

**Keywords:** neurological signs, first-episode psychosis, clinical outcome, remission

## Abstract

Minor neurological signs are subtle deficits in sensory integration, motor coordination, and sequencing of complex motor acts present in excess in the early stages of psychosis. Still, it remains unclear whether at least some of these signs represent trait or state markers for psychosis and whether they are markers of long-term disease outcome of clinical utility. We examined the relationship between neurological function at illness onset assessed with the Neurological Evaluation Scale and subsequent illness course in 233 patients from AESOP-10 (Aetiology and Ethnicity in Schizophrenia and Other Psychoses), a 10-year follow-up study of a population-based cohort of individuals recruited at the time of their first episode of psychosis in the United Kingdom. In 56 of these patients, we also explored changes in neurological function over time. We included a group of 172 individuals without psychosis as controls. After 10 years, 147 (63%) patients had developed a non-remitting course of illness, and 86 (37%) a remitting course. Already at first presentation, patients who developed a non-remitting course had significantly more primary, motor coordination, and total signs than both remitting patients and healthy controls. While Motor Coordination signs did not change over time, rates of Primary, Sensory Integration, and Total signs increased, independently of illness course type. These findings suggest that motor coordination problems could be a useful early, quick, and easily detectable marker of subsequent clinical outcome. With other motor abnormalities, a measure of motor incoordination could contribute to the identification of the most vulnerable individuals, who could benefit from targeted and more assertive treatment approaches.

## Introduction

Minor neurological signs are subtle deficits in sensory integration, motor coordination, and sequencing of complex motor acts present in excess in the early stages of affective and nonaffective psychoses, and even in drug-naïve patients.^1^ Still, it remains unclear whether these signs represent trait or state markers of psychosis and whether they are markers of long-term disease outcome of clinical utility.

Minor neurological signs are already present at illness onset with moderate to large effect sizes of impairment in patients and first-degree relatives with no psychosis^2^ and may even precede the onset of psychosis, suggesting that they have a neurodevelopmental origin. As such, they may represent illness traits and endophenotypes.^3,4^ This is further supported by evidence that although often described as “soft,” these signs have consistent brain morphometric and functional correlates, including the precentral and postcentral gyri, premotor and frontal gyri, inferior parietal lobule, temporal gyri and insula, thalamus and basal ganglia, and cerebellum.^5–10^ Crucially, recent studies have also shown that sensorimotor dysfunction, proposed to be a primary domain of psychosis, is associated with aberrant connectivity of sensorimotor areas,^11^ iron loadings in the left accumbens,^12^ and volumes of medulla oblongata and pons.^13^ Furthermore, multimodal imaging data have shown that gray matter volume alterations co-occur with aberrant brain activity in cortical and cerebellar systems subserving sensorimotor dynamics and psychomotor organization, pointing to a defined pathophysiological substrate for sensorimotor dysfunctions and contributing to their definition as potential biomarkers for psychosis.^14^

Still, whether neurological signs, and sensorimotor dysfunction in particular, at illness onset are a marker of long-term clinical and functional psychosis outcome remains to be established. Only 2 studies have explored their ability to predict outcome 10 years after illness onset, but neither evaluated their stability over time.^15,16^ In those, Cuesta and colleagues^15^ found that signs score 6 months after onset predicted poorer psychosocial functioning, while White and colleagues^16^ found that signs at onset just predicted service dependency and only in patients with schizophrenia or schizoaffective disorder they predicted functional outcome.

More studies have evaluated neurological signs in relation to shorter-term outcomes, with some also exploring their trajectories, with inconsistent results, possibly due to inclusion of small and heterogeneous clinical samples and use of different neurological scales and outcome criteria. The study with the longest follow-up evaluated neurological signs trajectories over 5 years in 17 first-episode psychosis patients, finding that, while total neurological signs did not change, baseline primitive reflexes predicted subsequent poorer functional outcome.^17^ Other, shorter longitudinal studies also found no change in signs over time in first-episode patients, although they reported higher rates in association with more negative symptoms,^18,19^ higher antipsychotic dose,^20^ or improvement in psychopathology.^21^ In contrast, others found an increase in signs, for example, in patients with a non-remitting illness over 2^20^ and 5 years.^22^ Finally, several have also described a decrease in signs after illness onset, with a meta-analysis reporting that 14 out of 17 studies observed a decrease in signs over time in parallel with symptomatic remission.^23^ This was replicated recently in a large study of 349 patients, where a reduction in signs over 1 year correlated with an improvement in psychopathology and functioning, and rates at baseline predicted lack of treatment response.^24^ Of note, several studies reported that such reductions are more evident for sensorimotor signs^21,25^ in association with better clinical outcomes at 1 year^25^ and 6 months,^26^ and even in children and adolescents with early onset psychosis.^27^

Taken together, these findings confirm the significance of neurological signs, and particularly of sensorimotor deficits as one of the core dimensions of psychosis.^28^ Motor signs are easily quantifiable, and their potential link with illness outcomes makes them useful candidate markers for the prediction of long-term trajectories of psychosis course.

In this study, we examined the relationship between multiple domains of neurological function at psychosis onset and 10-year outcomes. We additionally examined change in signs during this time in a subset of these patients. To our knowledge, this is the first, large longitudinal study (*n* = 233 patients) to examine neurological function at first episode of any psychosis across multiple functional domains and over a long duration of illness. We hypothesized that higher motor neurological signs at onset and their persistence over time would be associated with a non-remitting illness course over the first 10 years of illness, reflective of their potential neurodevelopmental origin and of their role as early markers of long-term illness severity.

## Methods

This study was conducted as part of the Aetiology and Ethnicity in Schizophrenia and Other Psychoses (AESOP-10) study, a 10-year follow-up of a cohort of 557 patients, who consecutively presented to secondary mental health services in South East London, Nottingham, and Bristol (United Kingdom) for the first episode of a functional psychotic illness (International Classification of Disease [ICD-10] F10–19; F20–29; and F-30–39, psychotic coding).^29^ A detailed overview of the follow-up procedures in the AESOP-10 study has been published elsewhere.^30,31^ The study also recruited a group of 172 individuals without psychosis, aged 16–64 years, as a control group. All controls were screened for the presence of psychotic symptoms with the Psychosis Screening Questionnaire^32^ and excluded if they rated positively. All participants gave written consent and ethical approval was granted by the local Ethical Committee.

### Baseline Assessment

At baseline, sociodemographic and clinical information were obtained for all subjects. Handedness was assessed with the Annett Hand Preference Questionnaire.^33^ Premorbid Intelligence Quotient (IQ) was estimated with the National Adult Reading Test (NART)^34^ and current full-scale IQ was assessed using a shortened form of revised Wechsler Adult Intelligence Scale (WAIS-R). For patients, diagnoses were made according to the ICD-10 Criteria^29^ using the Schedules for Clinical Assessment in Neuropsychiatry (SCAN)^35^ based on consensus meetings with senior clinicians. Age of illness onset was evaluated with the Personal and Psychiatric History Schedule^36^ based on interviews with the patient, a close relative, and clinical notes. Duration of untreated illness was defined as the period in weeks from the onset of psychotic phenomena to first contact with statutory mental health services. Duration of illness was then defined as the time between onset of symptoms and time of assessment. Antipsychotic type and dose (in chlorpromazine equivalents) were also recorded.

### Follow-up Assessment and Illness Course

Information on course of illness and symptom history were obtained retrospectively at follow-up using an extended version of the World Health Organization (WHO) Life Chart^37^ based on case notes and clinical interview with patients and treating clinicians whenever possible. In interviews, we used significant anchor dates to assist recall and, as appropriate, interviews were structured around key events, such as hospital admissions. The SCAN^35 ^criteria were used to establish the absence or presence of psychotic symptoms over the follow-up period, consistent with WHO and other long-term outcome studies.^38^ Following the Schizophrenia Working Group Remission criteria,^39^ we adopted a 6-month period for establishing remission on the basis of absence of overt psychotic symptoms (operationalized as score of 2 or 3 on Rating Scale 2 in the SCAN; 0 = absence, 1 = symptom occurred, but fleeting, 2 = symptom definitely present, 3 = symptom present more or less continuously). From the WHO Life Chart, we used illness course type to classify patients into “non remitting,” those with a more severe course (in the Life Chart, those with a continuous or intermediate illness course, the former defined as having no periods of symptom remission greater than 6 months, and the latter as having at least one illness episode and one period of remission greater than 6 months) or “remitting,” those with a more benign course (in the Life Chart, those with an episodic illness course, defined as having one or more periods of remission greater than 6 months and no episode of psychosis, including the first one, lasting 6 months or more). Level of functioning was assessed using the Global Assessment of Functioning (GAF),^40^ which rates psychological, social, and occupational functioning. We recorded the number of weeks of treatment with antipsychotics from starting to stopping medications across the whole follow-up through face-to-face interviews and evaluation of patients’ records. We also estimated the proportion of the time patients could be considered adherent to medications over the follow-up using clinical notes and the WHO Life Chart.

### Neurological Signs

Neurological function was assessed at baseline and at follow-up with the expanded and previously validated version of Neurological Evaluation Scale (NES).^41,42^ For individuals with first-episode psychosis, the first assessment of neurological abnormalities was performed as soon as possible after initial presentation. The expanded version of NES is a structured scale providing scores in 4 subscales reflecting different functional areas and demonstrating good construct validity^41,43^: (1) primary neurological dysfunction (dysfunction that can be identified by a standard neurological examination); (2) sensory integration dysfunction (dysfunction apparent in the integration of sensory information); (3) motor coordination dysfunction (reflecting signs of motor incoordination); (4) motor sequencing dysfunction (reflecting the ability to perform complex motor sequences).

Scores for the items present in the original NES (included in the 3 subscales Sensory Integration, Motor Coordination, and Motor Sequencing) were left unchanged (from 0 = no abnormality to 2 = marked impairment, except for the snout and suck reflexes, scored as either 0 or 2). The remaining items (in the Primary signs subscale), were scored as 0 = no abnormality; 1 = intermediate criterion; 2 = clearly abnormal/marked impairment.^42^ Minor neurological signs were rated by physicians blind to diagnosis reaching a good interrater reliability (*r* = .87 to .96). Each subscale was analyzed separately in order to provide a better representation of the different neurological dysfunction than the global score. We evaluated extrapyramidal symptoms with the Simpson–Angus Rating Scale,^44^ akathisia with the Barnes rating scale,^45^ and tardive dyskinesia with the Abnormal Involuntary Movement in Schizophrenia Scale (AIMS).^46^

### Statistical Analysis

Descriptive data are presented as individual values, mean ± SD. Sociodemographic and neurological signs differences between groups were compared using unpaired *t*-test, ANOVA, or chi-square test as appropriate. We repeated these comparisons of neurological function using ANCOVA to account for potential confounders, including age, sex, ethnicity, and IQ. To estimate changes in neurological signs over time, we performed a repeated-measure ANOVA in individuals with both a baseline and a follow-up assessment. Correlation analyses were used to investigate correlations between factors. A hierarchical linear regression was used to explore whether neurological signs at baseline predicted functioning at follow-up over and above employment status at baseline. All statistical tests were 2-tailed. Statistical analyses were carried out using the Statistical Package for Social Sciences version 23.

## Results

For 233 patients who had a baseline neurological assessment, there were also data on illness course. Of these, 147 (63%) fulfilled criteria for a non-remitting course of illness, and 86 (37%) for a remitting course. Demographic and clinical characteristics of both patient groups and healthy controls are shown in table 1. The non-remitting group included significantly more males than the remitting and control groups. Both psychosis groups were slightly younger, less likely to be White British, and had a lower premorbid and full-scale IQ than controls. Non-remitting patients also had a significantly lower premorbid and full-scale IQ than remitting patients (table 1). Finally, the non-remitting group included a higher proportion of individuals with a diagnosis of schizophrenia, had more positive and negative symptoms and less hypomanic symptoms at baseline, and had significantly lower GAF-s and GAF-d scores at follow-up than the remitting group (table 1).

**Table 1.** Demographic and clinical characteristics of patients and controls

Characteristic	Non-remitting patients *n* = 147	Remitting patients *n* = 86	Controls *n* = 172	*P* (*t*-test/ANOVA/*x*^2^)
Female gender, *n* (%)	58 (40)	47 (55)	91 (53)	.02 (*x*^2^ = 7.5; *df* = 2)^a^
Age years, median (interquartile range)	29 (21–38)	28.5 (24–38)	35 (27–47)	<.00^b^ (*F* = 11.3; *df* = 2)
Handedness, *n* (% right)^c^	130 (89)	80 (93)	154 (91)	NS (*x*^2^ = 1.1; *df* = 2)
Ethnicity, *n* (%):				<.001^d^ (*x*^2^ = 21.1; *df* = 2)
White British	79 (54)	54 (63)	134 (78)	
Black and Minority ethnic	68 (46)	32 (37)	38 (22)	
Premorbid IQ, mean NART (SD)^e^	94. 91 (14.12)	101.78 (13.97)	106.74 (11.95)	<.001 (*F* = 26.8; *df* = 2)
Current full-scale IQ, mean WAIS-R (SD)^f^	85.95 (14.39)	95.92 (17.32)	105.30 (14.61)	<.001 (*F* = 55.0; *df* = 2)
Duration of untreated illness, weeks median (interquartile range)^g^	21 (5–71)	3 (1–6)	—	<.001 (*t* = 9.0; *df* = 204)
Duration of illness, weeks median (interquartile range)^h^	39 (17–94)	14 (9–29)	—	<.001 (*t* = 5.1; *df* = 195)
Lifetime diagnosis, *n* (%):			—	<.001^i^ (*x*^2^ = 37.3; *df* = 2)
Schizophrenia	82 (56)	16 (19)		
Affective psychosis	34 (23)	51 (59)		
Other psychosis	31(21)	20 (23)		
SCAN symptoms, mean (SD)^j^				
Positive	6.41 (4.43)	4.51 (3.47)		.001 (*t* = 3.3; *df* = 167)
Depressive	1.43 (2.08)	1.22 (1.52)		NS (*t* = 0.7; *df* = 195)
Hypomania	0.82 (1.54)	2.45 (2.69)		<.001 (*t* = −4.6; *df* = 91)
Negative	0.55 (0.73)	0.25 (0.53)		0.001 (*t* = 3.31; *df* = 176)
Total	11.79(6.07)	10.26 (5.41)		NS (*t* = 1.7; *df* = 195)
Negative symptoms during follow-up, *n* (%)^k^	49 (18)	5 (6)	—	<.001 (*x*^2^ = 21; *df* = 1)
Antipsychotics at baseline assessment, *n* (%)^l^			—	NS (*x*^2^ = 3.9; *df* = 3)
First generation	61 (50)	35 (47)		
Second generation	38 (31)	19 (26)		
Both first and second generation	2 (2)	0 (0)		
Drug naïve or drug free	21 (17)	20 (27)		
Chlorpromazine equivalents at baseline assessment, mean (SD)	185.3 (167.5)	174 (196.8)	—	NS (*t* = 0.4; *df* = 168)
Weeks on antipsychotics during follow-up, mean (SD)	287.1 (200)	153.8 (210)		<.001 (*t* = 3.8; *df* = 147)
Time adherent to medications over follow up (*n*, %)			—	NS (*x*^2^ = 3.6; *df* = 2)
0–33%	20 (19)	5 (8)		
34–67%	24 (22)	13 (21)		
68–100%	64 (59)	43 (71)		
GAF-s, mean (SD)^m^	55.29 (18.52)	74.59 (12.7)	—	<.001 (*t* = −8.4; *df* = 178)
GAF-d, mean (SD)	51.91 (17.46)	71.63 (15.65)		<.001 (*t* = −7.5; *df* = 176)

*Note:* GAF, Global Assessment of Functioning; IQ, intelligence quotient; NART, National Adult Reading Test; NS, not significant; SCAN, Schedules for Clinical Assessment in Neuropsychiatry; WAIS-R, Wechsler Adult Intelligence Scale.

^a^Post hoc analysis: non-remitting individuals had a significantly lower percentage of females than remitting individuals and controls (*P* = .04 and *P* = .02, respectively).

^b^Post hoc analysis: controls were significantly older than non-remitting (*P* < .001) and remitting (*P* = .001) individuals. There were no age differences between non-remitting and remitting individuals.

^c^Information on handedness was obtained for 146 people in the non-remitting group, 86 in the remitting group, and 169 controls.

^d^Post hoc analysis: controls had significantly more individuals of white ethnicity compared to non-remitting (*P* < .001) and remitting (*P* = .015) individuals.

^e^Information on NART IQ was obtained for 109 people in the non-remitting, 67 people in the remitting group, and 164 controls. Post hoc analysis: controls had a significantly higher NART IQ than remitting (*P* = .025) and non-remitting individuals (*P* < .001). Moreover, non-remitting individuals had a significantly lower IQ than remitting individuals (*P* < .002).

^f^Information on WAIS-R IQ was obtained for 114 non-remitting individuals, 70 remitting individuals, and 162 controls. Post hoc analysis: controls had a significantly higher total IQ than remitting (*P* < .001) and non-remitting individuals (*P* < 0.001). Furthermore, non-remitting individuals had a significantly lower IQ than remitting individuals (*P* < .001).

^g^Information on duration of untreated illness was obtained for 145 non-remitting individuals and 83 remitting individuals. The distribution of duration of untreated illness was highly skewed and, therefore, logarithmic transformation was used to compare it across the 2 groups using a parametric test.

^h^Information on duration of illness was obtained for 123 non-remitting individuals and 74 remitting individuals. The distribution of duration of untreated illness was highly skewed and, therefore, logarithmic transformation was used to compare it across the 2 groups using a parametric test.

^i^Post hoc analysis: the non-remitting group included more individuals with a diagnosis of schizophrenia (*P* < .001) and other psychosis (*P* = .005) than the remitting group.

^j^Symptom details were missing for 18 non-remitting individuals and 18 remitting individuals.

^k^Data on the presence of negative symptoms during the follow-up period were available for 141 non-remitting individuals and for 82 remitting individuals.

^l^Information on antipsychotic medications at baseline neurological evaluation was available for 196 patients.

^m^GAF-s scores were available for 118 non-remitting individuals and 68 remitting individuals, and GAF-d scores for 114 non-remitting individuals and for 64 remitting individuals.

Approximately 10 years later (mean 9.1 years, SD 2.1), we obtained a second neurological assessment in 56 patients. These individuals were similar to those who only had a baseline assessment in premorbid IQ (NART 99 vs 97, respectively) and illness course (remitting 36% vs 37%, respectively) but were slightly younger (28 vs 32 years of age), less likely to be White British (48% vs 60%), more likely to be males (62% vs 52%), and to have a diagnosis of nonaffective psychosis (62% vs 46%). Healthy controls were assessed only once at baseline.

### Relationship Between Baseline Neurological Signs Scores and Illness Course

At baseline, individuals who subsequently became non-remitting already showed significantly more neurological signs than both remitting individuals and controls. The one-way ANOVA identified significant between-group differences for Primary (*F* = 15.3; *df* = 2; *P* < .001), Motor Coordination (*F* = 48.1; *df* = 2, *P* < .001), Motor Sequencing (*F* = 5.4; *df* = 2; *P* = .005), and Total signs (*F* = 27.1; *df* = 2; *P* < .001). The post hoc analysis revealed that this was due to the non-remitting group showing higher scores than the remitting group for Primary (*P* = .030), Motor Coordination (*P* = .001), and Total signs (*P* = .001) than the remitting group. Furthermore, the non-remitting group also showed higher scores than controls for Primary (*P* < .001), Motor Coordination (*P* < .001), Motor Sequencing (*P* = .003), and Total signs (*P* < .001). In contrast, individuals who became remitting had more signs than controls on Motor Coordination (*P* < .001) and Total signs (*P* = .020). There were no differences in antipsychotic side-effects (table 2).

**Table 2.** Neurological signs and side-effect scales mean scores at baseline

Scale	Non-remitting patients *n* = 147	Remitting patients *n* = 86	Controls *n* = 172	Statistical significance
Neurological signs, mean (SD); (quartiles)				
Primary	3.9 (4.0); (1 3 6)	2.8 (2.9); (0 2 4)	2.0 (2.2); (0 1 3)	<.001 (*F* = 15.3; *df* = 2)
Sensory Integration	1.5 (1.9); (0 1 2)	1.1 (1.4); (0 0 2)	1.3 (1.5); (0 1 2)	NS (*F* = 1.9; *df* = 2)
Motor Coordination	2.6 (2.8); (0 2 4)	1.6 (1.8); (0 1 3)	0.4 (0.9); (0 0 0)	<.001 (*F* = 48.1; *df* = 2)
Motor Sequencing	2.2 (2.4); (0 2 4)	1.7 (2.1); (0 1 3)	1.5 (1.7); (0 1 2)	.005 (*F* = 5.4; *df* = 2)
Total	10.2 (8.2); (5 9 15)	7.3 (5.7); (3 6 10)	5.1 (3.9); (2 4 8)	<.001 (*F* = 27.1; *df* = 2)
Tardive dyskinesia, mean AIMS (SD)	0.7 (2.2)	0.6 (1.6)	—	NS (*t* = 0.4; *df* = 220)
Akathisia, mean Barnes (SD)	1.3 (2.3)	1.5 (2.7)	—	NS (*t* = −0.6; *df* = 221)
Extrapyramidal symptoms, Simpson–Angus mean (SD)	2.2 (3.3)	1.3 (1.6)	—	.008 (*t* = 2.7; *df* = 218)

*Note:* AIMS, Abnormal Involuntary Movement in Schizophrenia Scale; NS, not significant.

To ensure that differences in neurological function between groups were not influenced by age, gender, ethnicity, or IQ (premorbid and current), we performed a 2-way ANCOVA analysis using group membership, gender, and ethnicity as fixed factors and age and IQ as covariates. This analysis showed that there was still a significant effect of group for Primary signs (*F* = 10.4; *df* = 2; *P* < 0.001), Motor Coordination (*F* = 27.7; *df* = 2; *P* < 0.001), and Total signs (*F* = 14; *df* = 2; *P* < 0.001). The post hoc analysis confirmed that even when these factors were taken into account, the non-remitting group still had more Primary, Motor Coordination, and Total neurological signs at baseline than both remitting individuals (*P* = .020, *P* = .003, and *P* = .002, respectively) and healthy controls (*P* < .001). As there were more patients with a diagnosis of schizophrenia in the non-remitting group, we compared neurological signs between patients with schizophrenia, affective psychoses, and other psychoses and found no significant differences in mean scores between groups (all *P* > .2). This suggests that it is unlikely that the higher neurological signs in non-remitting patients were due to the higher proportion of patients with schizophrenia in this group.

Finally, we conducted an additional exploratory hierarchical regression analysis to examine neurological signs ability to also predict functional outcome (GAF-d scores) over and above baseline employment status (a proxy measure of baseline functioning). Employment status at baseline accounted for 15.1% (*R*^2^ = .151; *P* < .001) of the variation in GAF score at follow-up, and adding total neurological signs to the model increased variance to 17.7% (*R*^2^ = .177; delta *R*^2^ = .026, *F* = 5.56, *F* change *P* = .019), suggesting that neurological signs at presentation are also predictive of functional and not only clinical outcomes.

### Longitudinal Changes in Neurological Signs Over the First 10 Years of Illness

The neurological signs scores for the 56 subjects who completed the 2-time points neurological evaluations are presented in table 3. Of these, 36 (64%) were classified as non-remitting, and 20 (36%) were classified as remitting. To assess changes in neurological signs over the first 10 years of illness, we performed a repeated measure ANOVA with time as within-subject factor and group (non-remitting and remitting) as between-subject factor. Both non-remitting and remitting groups showed an increase in score over time for Primary (time effect *F* = 8.5; *df* = 1; *P* = .005), Sensory Integration (time effect *F* = 5.9; *df* = 1; *P* = .02) and Total signs (time effect *F* = 7.9; *df* = 1, *P* = .007). No significant change over time was observed for Motor Coordination signs in the 2 groups (time effect *F* = 0.07; *df* = 1; *P* = .1; time × group effect *F* = 0.17; *df* = 1; *P* = .6). Interestingly, there was a nonsignificant increase in Motor Sequencing signs only in the non-remitting group (time × group effect *F* = 4.9, *df* = 1, *P* = .031). There were no between-group or time differences in the course of Barnes, Simpson–Angus, or AIMS mean scores.

**Table 3.** Neurological signs and side effect scales mean scores at baseline and at follow-up in patients with non-remitting and remitting course of illness (ANOVA)

Scale, mean (SD)	Non-remitting patients *n* = 36		Remitting patients *n* = 20		Time effect	Time × group effect
	Baseline	Follow-up	Baseline	Follow-up	*P* (*F*; *df*)	*P* (*F*; *df*)
Primary	4.4 (3.7)	7.6 (6.5)	3.9 (3.6)	6.0 (4.6)	**0.005** (8.5; 1)	NS (0.3; 1)
Sensory Integration	1.6 (1.8)	2.8 (2.2)	0.7 (1.0)	1.1 (1.6)	**0.02** (5.9; 1)	NS (1.3; 1)
Motor Coordination	1.9 (2.0)	1.7 (2.3)	0.8 (1.1)	0.9 (1.0)	NS (0.07; 1)	NS (0.17; 1)
Motor Sequencing^a^	2.1 (2.3)	3.9 (3.5)	1.2 (1.9)	1.1 (1.3)	NS (3.5; 1)	**0.031** (4.9; 1)
Total	10.2 (7.3)	15.3 (11.0)	6.5 (6.0)	9.0 (5.5)	**0.007** (7.9; 1)	NS (0.96; 1)
Tardive dyskinesia, AIMS^a^	0.21 (0.5)	0.37 (1.6)	0.47 (0.91)	0.07 (0.26)	NS (0.3;1)	NS (1.6;1)
Akathisia, Barnes^a^	1.2 (2.3)	2.1 (2.7)	1.1 (2.0)	0.7 (1.8)	NS (0.23;1)	NS (1.9;1)
Extrapyramidal symptoms, Simpson–Angus^a^	2.3 (2.8)	3.9 (7.1)	1.1 (1.1)	0.6 (1.4)	NS (0.3; 1)	NS (1.0; 1)

*Note:* AIMS, Abnormal Involuntary Movement in Schizophrenia Scale; NS, not significant; bold *P* values indicate significance values <.05.

^a^Motor sequencing score for missing for 1 patient; AIMS scores were available for 40 patients, Barnes scores for 45 patients, and Simpson–Angus scores for 38 patients.

### Relationship With Antipsychotic Medications

We explored the potential role of antipsychotic medications in explaining neurological signs rates. We found no differences in the proportion of non-remitting and remitting patients taking first- or second-generation antipsychotics, being drug-free or naïve at baseline, nor in antipsychotic dose (table 1). Furthermore, there was no correlation between baseline antipsychotic dose and baseline neurological signs or between number of weeks on medications and neurological signs at follow-up (table 4). This suggests that between-group differences in baseline neurological signs and change in signs over time are unlikely to be related to antipsychotic exposure.

**Table 4.** Correlations between neurological signs scores and antipsychotics

Correlation between baseline neurological signs scores and chlorpromazine equivalents (mg)	Pearson *r*	Significance *P*
Primary signs	.073	.34
Sensory Integration signs	.05	.48
Motor Coordination signs	.07	.38
Motor Sequencing signs	−.05	.50
Total signs	.06	.47
Correlation between follow-up neurological signs scores and time on antipsychotics during the follow-up (weeks)	Pearson *r*	Significance *P*
Primary signs	.065	.65
Sensory Integration signs	.23	.1
Motor Coordination signs	−.05	.74
Motor Sequencing signs	.005	.97
Total signs	.004	.79

## Discussion

To the best of our knowledge, this is the first prospective study that has investigated the relationship between different domains of neurological signs at illness onset and long-term illness course and functional outcome, as well as change over the first 10 years of illness in a large sample of individuals with first-episode psychosis. Our main finding is that, at their first presentation to services, those patients who subsequently develop a more severe illness course (non-remitting) already show significantly more Primary, Motor Coordination, and Total signs than both those who follow a more favorable course (remitting) and healthy controls, even when correcting for potential confounders, such as cognitive ability. This suggests that certain domains of neurological impairment represent early markers of a more severe illness type. Our second main finding is that, independently of illness course type, impairments in Motor Coordination remain stable over time and represent a trait-like feature of psychosis, while deficits in Primary, Sensory Integration, and Total signs worsen, suggesting they reflect illness duration.

This study advances our previous findings that, at first presentation, only Primary and Motor Coordination signs are specific to the presence of psychosis,^47^ showing that their higher rates at onset characterize those patients who develop a poorer illness course with worse functioning, possibly reflecting a more severe pathophysiological process. In addition, we find that Motor Coordination signs remain stable over time, likely representing illness traits. Indeed, this is consistent with reports from shorter follow-ups that motor signs change little over 1, 2, 3, and 5 years and even across the lifespan in patients with schizophrenia,^18,48–50^ that they predict poorer functional outcomes,^51^ and that distinct sensorimotor performance profiles may be even markers of risk for psychosis.^52^ Motor coordination with abnormal involuntary movements and catatonia could represent a sensorimotor dimension that cuts across psychopathology and that has etiological and prognostic value as a psychosis endophenotype.^28,53^ As such, sensorimotor function became one of the Research Domain Criteria (RDoC) in 2019. As neurological soft signs reflect the integration of multiple rather than focal processes, they could well be considered abnormalities in any of the sensorimotor RDoC subconstructs, involving action planning, initiation, sensorimotor dynamics, execution, or inhibition. The recognition of sensorimotor dysfunction could help the identification and stratification of patients based on an objective, quantifiable measure that could inform clinical management and advance the investigation of the neurobiological correlates of psychoses subtypes.

In this regard, our group and others have shown that sensorimotor coordination deficits represent the expression of underlying cortical and subcortical brain alterations specifically associated with the pathophysiology of psychosis.^6,28^ Furthermore, while worse motor coordination has been linked with anterior cingulate-caudate aberrant connectivity, catatonia and dyskinesia have been associated with thalamocortical connectivity, suggesting that sensorimotor abnormalities are linked to multiple pathophysiological mechanisms.^54^ Finally, recent evidence that volume alterations co-occur with aberrant brain activity in cortical and cerebellar systems subserving sensory- and psychomotor organization adds to the concept of sensorimotor dysfunctions as potential biomarkers for psychosis.^14^ As such, it is not surprising that motor coordination problems would be associated with more severe manifestations of the disease.

While there are no previous long-term studies that have investigated subgroups of neurological signs, our findings are consistent with only 2 studies with a 10-year follow-up, which showed that total neurological signs predict poorer psychosocial functioning,^15^ particularly in patients with schizophrenia,^16^ and service dependency.^16^ Similar evidence also comes from shorter and medium-term follow-ups of first-episode psychosis patients. For example, a decrease in neurological signs over the first year of illness has been associated with better clinical^24,25^ and functional outcomes^24^ and with less prominent negative symptoms, a potential indicator of illness severity.^19^ Furthermore, higher baseline neurological signs at onset have also been found to predict lack of treatment response as early as 6 weeks^55^ and even 1 year after illness onset.^15^ Finally, even when evaluated before illness onset, neurological signs in individuals at ultra-high risk of psychosis have been found to predict illness transition to the illness, as well as the onset of severe negative symptoms 12 months later.^4^

Another interesting finding in our study is that rates of sensory integrative signs at baseline do not differ between non-remitting and remitting groups, nor from healthy controls. This advances our previous report of a similar sensory integration performance in patients with psychosis and controls, showing also that these signs are not markers of subsequent illness course and that they increase over time in both patients groups, possibly reflecting aging rather than illness severity.^47,56^ Indeed, this may also be the case for Primary signs, which we found to also increase over time in both remitting and non-remitting patients.

In contrast, only Motor Sequencing signs increased over time in patients with a non-remitting course. It is difficult to interpret this finding, which is consistent, at least in part, with reports that an increase or lack of change in function is present in patients with worse outcomes and a reduction in those with a more episodic course.^23,57^ In fact, in the few studies that reported an overall increase in neurological impairment after a first psychotic episode, this increase was mostly seen in the subgroup of patients with a worse clinical course.^20,22,58^ Taken together, these findings support the hypothesis that different sensorimotor signs represent a combination of trait- and state-like illness features.

Critical to the interpretation of neurological and particularly sensorimotor signs in psychosis is the evaluation of their relationship with antipsychotic use. Our findings, however, confirm previous reports from us and others^2,47,59^ that sensorimotor abnormalities represent an intrinsic feature of psychoses and are not related to medications use.^53^ Here, we evaluated neurological signs at baseline, prior to long-term exposure to antipsychotics, and found no differences in the type or dose of antipsychotic in non-remitting and remitting patients, nor any correlation between antipsychotic exposure and neurological performance at the 10-year follow-up. This makes our results even stronger, showing that differences in neurological performance between patients who develop different illness trajectories are unlikely to result from differences in antipsychotic treatment.

The present study has several strengths, particularly the large sample size with neurological function evaluated at baseline and the long follow-up period. The evaluation of clinical outcome over a long period of time, with a standardized instrument allowed us to investigate neurological function as an early marker of poor long-term illness outcome. Although we could evaluate changes in neurological function only in a subset of patients, we provide here the first report of long-term changes that follow the first episode of psychosis. This should be evaluated in larger clinical populations in parallel with other illness indicators. We cannot exclude the possibility of selection bias as, in longitudinal studies, those patients more likely to be traced could be those with a more severe illness type and worse functional outcome. However, the sample with baseline neurological function and illness course data we included here was similar to the AESOP-10 core sample for age, sex, and illness course and included slightly less individuals with a diagnosis of schizophrenia.^31^ While, here, we had more patients with schizophrenia in the non-remitting group, as in our previous report,^47^ we found no differences in neurological function across diagnostic groups, suggesting that neurological signs are a marker of illness course rather than diagnosis. Finally, while psychotropic substances, such as cannabis or amphetamines, could also affect neurological function, the proportion of patients with lifetime cannabis or amphetamines use was similar in the non-remitting and remitting group (34% vs 37% for amphetamines and 88% vs 86% for cannabis, respectively), making it unlikely that substance use patterns could explain motor coordination signs differences between groups.

In conclusion, we suggest that some neurological abnormalities, specifically motor coordination problems, could be a useful early, quick, and easily detectable indicator of subsequent clinical outcome that could help stratify patients early in the illness, identifying those who might benefit from a particularly assertive treatment. From a therapeutic perspective, the presence of sensorimotor abnormalities could also inform the choice of antipsychotics, including consideration for clozapine as appropriate, given its possible beneficial effects on sensorimotor dysfunction and favoring an approach focused not only on symptomatic improvement but also on limiting sensorimotor abnormalities that may hamper social functioning in the community.

## Funding

This work was supported by the UK Medical Research Council (ref: G0500817) and the Department of Health via the National Institute for Health Research (NIHR) Biomedical Research Centre at South London and Maudsley NHS Foundation Trust and King’s College London. According to UK research councils’ Common Principles on Data Policy, we are working toward making data supporting this study available.
